# Impact of *Torulaspora delbrueckii* During Fermentation on Aromatic Profile of Vidal Blanc Icewine

**DOI:** 10.3389/fmicb.2022.860128

**Published:** 2022-06-07

**Authors:** Jing Li, Mengnan Hong, Baiyu Qi

**Affiliations:** Institute of Food Science and Engineering, Jinzhou Medical University, Jinzhou, China

**Keywords:** indigenous icewine yeast, *Torulaspora delbrueckii*, mixed culture fermentation, Vidal blanc icewine, aroma compounds, HS-SPME-GC-MS

## Abstract

Non-*Saccharomyces* yeasts usually have a positive effect on improving the diversity of wine aroma and increasing the differentiation of wine products. Among these non-*Saccharomyces* yeast species, *Torulaspora delbrueckii* is often studied and used in winemaking in recent years, but its application in icewine has not been reported yet. In this study, indigenous *T. delbrueckii* strains (TD1 and TD2) and *Saccharomyces cerevisiae* strains (commercial yeast SC1 and indigenous icewine yeast SC2) were sequentially inoculated for icewine fermentations; meanwhile, pure *S. cerevisiae* (SC1 and SC2) fermentations were used as the control; TD1, TD2, and SC2 strains used were screened from spontaneous fermentations of Vidal blanc icewine. The aim was to investigate the effect of *T. delbrueckii* on the aroma complexity of icewine, which is of great significance to the application of *T. delbrueckii* in icewine production. The results showed that *T. delbrueckii* was completely replaced by *S. cerevisiae* at the middle and later fermentative stages in mixed culture fermentations. Compared with the icewine fermented with pure *S. cerevisiae*, mixed culture fermented icewines contained lower acetic acid and ethanol, and higher glycerol. The inoculation of *T. delbrueckii* greatly impacted the levels of several important volatile compounds, and more 2-phenylethyl alcohol, isoamyl acetate, linalool, D-limonene, p-cymene and cineole were produced, and the fruity, flowery, and sweet characteristic was intensified. Moreover, the relevance of strain-specificity within *T. delbrueckii* to aroma compound differences was shown. To our knowledge, this study is the first to investigate the application of *T. delbrueckii* in Vidal blanc icewine fermentation, and volatile aroma compounds in the icewine fermented by *T. delbrueckii* and *S. cerevisiae*.

## Introduction

Icewine is a type of dessert wine that is fermented from juice squeezed from naturally frozen grapes (Lan et al., 2019). Ice grape juice generally contains high soluble solids level (>35°Bx). Icewine is characterized by high levels of titratable acid (>6.5 g/L, represented as tartaric acid), residual sugar (>125 g/L), as well as aromatic compounds (Bowen and Reynolds, 2015). Because of the specific climatic conditions required for planting and harvesting ice grapes, icewine is only produced in a few countries, such as Canada, Germany, Austria, China, and the United States (Alessandria et al., 2013; Bowen and Reynolds, 2015). In recent years, the Chinese icewine industry has developed rapidly, and China has become an icewine producer that cannot be ignored (Huang et al., 2018). In China, Huanren is a major region that produces icewine, and Vidal blanc is the main variety of grapes for icewine manufacturing in this region. Vidal blanc grapes are resistant to cold in winter because of their thick skin. Vidal blanc icewine is considered the highest quality icewine because of its appealing aroma and attractive flavor (Crandles et al., 2015). In addition to Vidal blanc, Riesling, Chenin Blanc, Chardonnay, Beibinghong are other common grape varieties used in icewine production (Lan et al., 2019).

Aroma is one of the most important aspect of wine quality, and the majority of aromatic compounds are formed during the fermentation process. Alcoholic fermentation of grape juice is a complex biochemical process carried out by dynamic microbiota, in which yeast plays an important role (Li et al., 2019). In the process of conversion of ice grape juice into icewine, the composition changes dramatically under the action of both *Saccharomyces* and non-*Saccharomyces* yeasts (Li et al., 2019). Several studies have shown that non-*Saccharomyces* yeasts positively contribute to the aroma and flavor of wine by secreting certain enzymes (such as esterases, β-glucosidase, β-xylosidase, and pectinase; Maturano et al., 2012; Hong et al., 2019). Therefore, in recent years, mixed culture of non-*Saccharomyces* yeast during fermentation is an emerging strategy for improving the wine aroma complexity, which has also gradually attracted attention from winemakers and enhanced market competitiveness (Lencioni et al., 2016; Padilla et al., 2016; Englezos et al., 2018). However, the level of current knowledge on the impact of non-*Saccharomyces* yeast on wine quality is still far from satisfactory. Indeed, some non-*Saccharomyces* species with application potential isolated from spontaneous fermentation of wine have not been studied enough, and knowledge about strain heterogeneity within a single non-*Saccharomyces* species and the effect of strain-specificity on wine aromas is still limited (Azzolini et al., 2015; Hong et al., 2021). At present, the studies regarding icewine mainly have focused on the aroma compounds and flavor characteristics of traditional icewine inoculated singly with *Saccharomyces cerevisiae* (Bowen and Reynolds, 2015; Huang et al., 2018; Lan et al., 2019). But, there are little information on the application of non-*Saccharomyces* yeast in icewine fermentation and the effects of the multiculture of non-*Saccharomyces* and *S. cerevisiae* strains on aroma profiles of icewine. The fermentation conditions of icewine are different from those of general wine, which are characterized by high sugar concentrations, high acid concentrations and low fermentation temperature (Zhang et al., 2018).

*Torulaspora delbrueckii* is a non-*Saccharomyces* species with excellent fermentation capacity, which is often studied and used in winemaking in recent years (Azzolini et al., 2015; Zhang et al., 2018). These studies revealed that mixed culture of *T. delbrueckii* during wine fermentation can decrease volatile acidity and acetaldehyde content, and increase fruity esters, higher alcohols and other positive aroma components (Loira et al., 2014; Velázquez et al., 2015; Belda et al., 2017). Moreover, *T. delbrueckii* was the first non-*Saccharomyces* species to be produced into active dry yeasts for winemaking, but the knowledge about *T. delbrueckii* strain heterogeneity and the impact of strain-specificity on wine aromas is also very little (Azzolini et al., 2015). In addition, the use of *T. delbrueckii* strains during icewine fermentation has not been found.

In recent years, the use of indigenous yeasts such as *Candida zemplinina*, *Hanseniaspora uvarum*, *Metschnikowia pulcherrima* to increase the complexity of wine aroma has gradually become a trend of winemaking with regional identities (Liu et al., 2016; Tofalo et al., 2016). In our previous study, we investigated that extensive yeast strains were isolated from Vidal blanc icewine during the spontaneous fermentation (Li et al., 2019), and then tolerance, and aroma-related enzymes activities of these yeast strains were studied in order to select the indigenous strains (including *T. delbrueckii* strains used in this study) with the potential to produce characteristic icewine (Hong et al., 2019). In this paper, based on the abilities of indigenous *T. delbrueckii* strains to adapt to the icewine micro-environment and their fermentation performances, we used them during fermentation and analyzed the aroma of the icewine. The main objective of this study was to reveal the effects of mixed culture of indigenous *T. delbrueckii* strains and *S. cerevisiae* (contrasted with pure culture of *S. cerevisiae*) during fermentation on aromatic profile of Vidal blanc icewine under laboratory-scale conditions.

## Materials and Methods

### Yeast Strains and Raw Ice Grape Juice

Three indigenous yeast strains were used in this study, two of which were *T. delbrueckii*, named as TD1 and TD2; the other strain was *S. cerevisiae*, named as SC2. TD1, TD2 and SC2 were isolated from the spontaneous fermentations of Vidal blanc icewines (Li et al., 2019). Moreover, commercial *S. cerevisiae* (ST, LAFFORT, Bordeaux, France) was also used, and named as SC1.

The GenBank accession numbers (ITS region) of TD1, TD2 and SC2 were MK318913, MK318908, and MK123424; TD1, TD2 and SC2 have been screened by tolerance experiments (ethanol, SO2, sugar, and acid concentration) and enzymatic activities experiments (β-glucosidase, β-xylosidase, and pectinase; Hong et al., 2019). These yeast strains were cultured in YPD medium (yeast extract 10 g/L, peptone 20 g/L, dextrose 20 g/L; Haibo, Qingdao, China) at 28°C for 48 h, and stored at −80°C after addition of glycerol (30%, v/v), as described by Alessandria et al. (2013).

The ice grape juice used for icewine fermentation in this study was taken from Milan winery (41°17′53.53′′N 125°22′27.26′′E) in January 2019. The raw ice grape juice had a pH of 4.03, reducing sugar 432.97 g/L, and total soluble solids 41.0°Bx.

### Pure and Mixed Culture Fermentations

Fifty milligrams per liter SO_2_ was added in the ice grape juice after squeezing, and the ice grape juice samples were heated at 70°C for 20 min to sterilize. Laboratory-scale fermentations were conducted in 250 ml sterile flasks filled with 180 ml of the sterilized ice grape juice, and all flasks were cultured at 18°C under static conditions for 30 days (Li et al., 2018). Pure and mixed cultured fermentations were carried out as follows: (1) single inoculation of SC1; (2) sequential inoculation with TD1 followed by SC1 after 48 h (TD1/SC1); (3) sequential inoculation with TD2 followed by SC1 after 48 h (TD2/SC1); (4) single inoculation of SC2; (5) sequential inoculation with TD1 followed by SC2 after 48 h (TD1/SC2); and (6) sequential inoculation with TD2 followed by SC2 after 48 h (TD2/SC2). Every experiment was set up in triplicate. Yeast strains were pre-activated at 28°C using YPD medium. The initial cell concentration was determined through optical density at 600 nm (OD_600_) and plate counts on Wallerstein Laboratory (WL) Nutrient medium (Englezos et al., 2016; Tronchoni et al., 2017). The initial cell concentrations of *T. delbrueckii* and *S. cerevisiae* in mixed culture fermentations were approximately 10^6^ cells/ml, with the inoculation ratio of 1:1. The initial cell concentration of *S. cerevisiae* was approximately 10^6^ cells/ml in the control fermentations.

### Sampling

Samples of longitudinal icewine fermentation were taken at 0, 2, 4, 7, 14, 21, and 30 days, and yeast population dynamic changes during icewine fermentation were monitored. Each sample was serially diluted in a sterile physiological solution (with ratios of 1:10–1:10^6^) and spread-plated on WL Nutrient agar (Haibo, Qingdao, China) that was used for colony counting and differentiating *T. delbrueckii* and *S. cerevisiae* by their different colony colors and morphologies (Cavazza et al., 1992; Li et al., 2018), and the plates were incubated at 28°C for 5 days. During pure and mixed culture fermentations, the fermentation kinetic was evaluated by monitoring the amount of CO_2_ produced every 24 h (the decrease in the weight of the flask; Lee and Park, 2020), until the weight loss of the flasks remained constant for 3 consecutive days. The mean values of CO_2_ daily production were recorded. At the end of fermentations, the samples of icewines were centrifuged and collected cell-free supernatants for analysis of aromatic compounds.

### Analysis of Major Chemical Components in Icewine

Residual sugars and titratable acids were determined by the O.I.V. Methods (OIV, 2010).

Organic acids and glycerol were quantified by using a Prominence LC-20A (SHIMADZU, Japan) system for high-performance liquid chromatography (HPLC). The filtered icewines (through 0.22 μm filter membrane) were analyzed by using the WondaSil C18-WR (250 mm × 4.6 mm, 5 μm) column. Determination of glycerol used a RID-10A refractive index detector (SHIMADZU, Japan). Both acids were detected at 210 nm. Column temperature was thermostated at 35°C for 10 min. The solvent A (980 ml/L 0.02 mol/L KH_2_PO_4_, pH adjusted to 2.0 with phosphoric acid) and the solvent B (20 ml/L acetonitrile) were used as the mobile phase at a flow rate of 0.8 ml/min. The injection volume was 10 μl. The standard curve was drawn according to the standard solution calculated by different concentration gradients, with standard concentration as abscissa and peak area as ordinate.

Ethanol content was determined by the Fuli Gas Chromatograph 9,790 Plus coupled with flame ionization detector (FID; Fuli Analytical Instrument Co., Ltd., Zhejiang, China). One microliter of each sample (prefiltered through 0.22 μm membrane) spiked with internal standard (1-propanol) was injected into KB-5 capillary columns (30 m × 320 μm × 0.25 μm, Kromat Corporation, Bordentown, United States). Nitrogen (99.999%) was used as a carrier gas (1.0 ml/min). Split injection was used with a split ratio of 50:1. The ignition was carried out with synthetic air (300 ml/min) and hydrogen (30 ml/min). Temperatures of injector and detector were both kept at 250°C. The oven temperature was from 45°C held for 5 min to 50°C at 5°C/min, then increased to 230°C at 20°C/min held for 2 min. GC quality standard reagents (Shanghai Aladdin Biochemical Technology Co., Ltd.) were used to calibrate the machine for the ethanol measured.

### Analysis of Major Volatile Aroma Compounds by Headspace Solidphase-Microextraction-Gas-Chromatography-Mass-Spectrometry

Volatile compounds were extracted by solid-phase-microextraction (Lan et al., 2016). A 5 ml icewine sample was mixed with 10 μl of 4-methyl-2-pentanol (internal standard, 1.0018 g/L) and 1 g NaCl, and placed in a 15 ml vial. The vial was covered with a PTFE-silicon septum and equilibrated at 40°C for 30 min on a heated magnetic stirrer with agitation at 300 rpm. Volatile compounds were analyzed using a headspace solid-phase micro-extraction (HS-SPME) coupled with DVB/CAR/PDMS 50/30 μm SPME fiber (supelco, Bellefonte, PA, United States) and detected by gas chromatography–mass spectrometry (GC–MS-QP2010, PLUS, Shimadzu, Kyoto, Japan), as described by Hong et al. (2021). A capillary column of 30 m × 0.25 mm × 0.25 μm RxiTM-5 ms (J & amp; WScientific Folsom, CA, United States) were used and with helium (99.999%) as the carrier gas at a rate of 1.0 ml/min. Injections were in split mode at 10:1. The temperature of the injection port, interface and ion source were 250°C, 230°C, and 230°C, respectively. The oven temperature was programmed at 35°C for 3 min and increased to 160°C at a rate of 6°C/min, then ramped to 250°C at a rate of 10°C/min. The mass spectrometer was in electron ionization (EI) mode at 70ev with the full scan mode (*m*/*z* 35–350). A total of 257 volatile compounds were detected, and the volatile aroma compounds were identified by comparing their mass spectrum (MS) in the NIST 05 library (compounds with a matching rate greater than 80% were retained) and comparisons of retention indices reported in the GCMS solution (version 2.6).

### Statistical Analysis

Data obtained from icewine fermentations was expressed as the mean ± SD. The Duncan test for *p* < 0.05 and one-way ANOVA were used to evaluate any differences among fermentations under the different strategies, using the SPSS statistical package version 17.0 (SPSS Inc., United States). Peak area of aroma compounds were normalized using “Autoscaling” (mean-centered and divided by the SD of each variable), and executed a bubble chart by an online platform for data analysis and visualization.^1^ MetaboAnalyst 2.0^2^ was used to do principal component analysis (PCA).

## Results

### Yeast Population Dynamic Changes During Pure and Mixed Culture Fermentations

**Figure 1:**
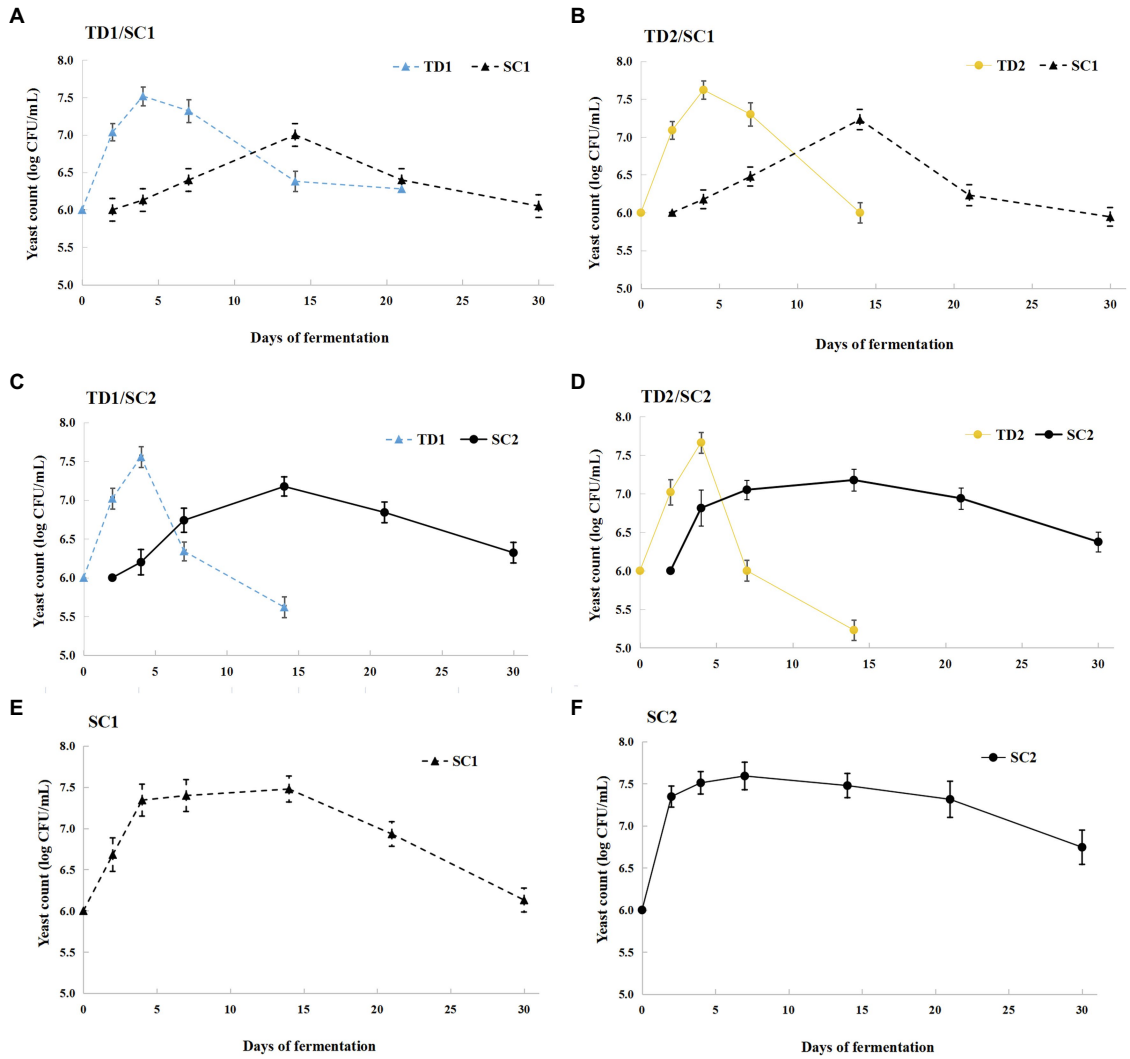
Yeast population dynamic changes during pure and mixed culture fermentations. TD1/SC1, TD2/SC1, TD1/SC2, TD2/SC2 **(A–D)**: sequential inoculation with *Torulaspora delbrueckii* (TD1, TD2) followed by *Saccharomyces cerevisiae* (SC1, SC2) after 48 h; SC1 and SC2: single inoculation of *S. cerevisiae* SC1 **(E)** and SC2 **(F)**, respectively.

The dynamics of *T. delbrueckii* and *S. cerevisiae* during the fermentation process are illustrated in Figure 1. During pure culture fermentations, the growth trend of *S. cerevisiae* increased first and then decreased. SC2 achieved maximum cellular concentration earlier than SC1; SC1 reached maximum cellular concentration (7.48 log CFU/ml) on day 14, while SC2 reached a peak (7.59 log CFU/ml) on day 7 (Figures 1E,F). During mixed culture fermentations, the cellular concentrations of *T. delbrueckii* increased first, achieved a maximum (7.52–7.66 log CFU/ml) on day 4, and then decreased, and were not detected in the later fermentative stage. The cellular concentrations of *S. cerevisiae* increased first and then decreased, and reached maximum cellular concentration approximately 7.2 log CFU/ml on day 14; *S. cerevisiae* dominated in the middle and later fermentative stages. The cellular concentrations of *T. delbrueckii* declined when inoculated with *S. cerevisiae* (SC1 and SC2) 48 h later; the cellular concentrations of SC1 exceed *T. delbrueckii* (TD1 and TD2) on day 14, while the cellular concentrations of SC2 exceed *T. delbrueckii* (TD1 and TD2) on day 7 (Figures 1A–D).

### Basic Chemical Parameters in Icewines

The basic chemical compositions of pure and mixed culture fermented icewines, including titratable acidity, succinic acid, tartaric acid, acetic acid, glycerol, residual sugar and ethanol are shown in Table 1. Titratable acidity concentration of all icewines ranged from 5.65 g/L (TD2/SC2) to 9.15 g/L (SC1), and the concentrations of titratable acid in mixed culture fermented icewines were lower than those of pure fermented icewines. Acetic acid concentration ranged from 1.04 g/L (TD2/SC2) to 2.02 g/L (SC1) which were lower than the maximum allowable value of 2.1 g/L (OIV, 2015). Compared to the control fermentations, acetic acid contents produced by mixed culture fermentations were lower. Glycerol concentration ranged from 10.80 g/L (SC1) to 12.02 g/L (TD2/SC2), and the concentrations of glycerol in mixed culture fermented icewines were higher than those of pure fermented icewines. The residual sugar concentration of all icewines ranged from 164.20 g/L (SC2) to 220.88 g/L (TD1/SC1); compared to the icewines fermented by *S. cerevisiae* alone, the icewines fermented by mixed culture fermentations with *T. delbrueckii* and *S. cerevisiae* had higher residual sugar contents. Moreover, TD1/SC1 icewine had the lowest ethanol concentration (9.39%) and SC2 icewine with single inoculation of had the highest ethanol concentration (12.93%); the ethanol concentrations of mixed culture fermentations with *T. delbrueckii* and *S. cerevisiae* were lower than those of the control fermentations. No significant differences were observed in succinic acid and tartaric acid levels in different icewines. Supplementary Figure S1 shows that the total CO_2_ productions by the control fermentation are higher than those of mixed culture fermentation.

**Table 1:** The main chemical compositions in mixed and pure culture fermented icewines.

	TD1/SC1	TD2/SC1	SC1	TD1/SC2	TD2/SC2	SC2
Titratable acidity (g/L)	7.93 ± 0.07^b^	6.52 ± 0.4^c^	9.15 ± 0.01^a^	6.33 ± 0.35^c^	5.65 ± 0.32^d^	8.92 ± 0.33^a^
Acetic acid (g/L)	1.28 ± 0.01^c^	1.05 ± 0.01^e^	2.02 ± 0.01^a^	1.14 ± 0.01^d^	1.04 ± 0^f^	1.47 ± 0.01^b^
Succinic acid (g/L)	1.62 ± 0.03^a^	1.58 ± 0.05^a^	1.48 ± 0.11^a^	1.55 ± 0.02^a^	1.47 ± 0.03^a^	1.44 ± 0.07^a^
Tartaric acid (g/L)	1.56 ± 0.19^b^	1.60 ± 0.04^a^	1.52 ± 0.10^a^	1.71 ± 0.12^a^	1.73 ± 0.04^a^	1.70 ± 0.05^a^
Glycerol (g/L)	10.85 ± 0.53^a^	10.96 ± 0.33^a^	10.80 ± 0.39^a^	11.97 ± 0.12^a^	12.02 ± 0.16^b^	11.21 ± 0.20^c^
Residual sugar (g/L)	220.88 ± 0.12^a^	218.39 ± 0.24^b^	192.76 ± 0.1^c^	186.88 ± 0.29^d^	183.58 ± 0.31^e^	164.20 ± 0.29^f^
Ethanol (% v/v)	9.39 ± 0.03^e^	9.53 ± 0.01^e^	11.53 ± 0^c^	11.17 ± 0.02^d^	12.14 ± 0.02^b^	12.93 ± 0.45^a^

*Values are expressed means ± SDs obtained from the triplicate fermentations. Different letters in the same row indicate significant differences (*p* < 0.05)*.

### Volatile Aroma Compounds

**Figure 2:**
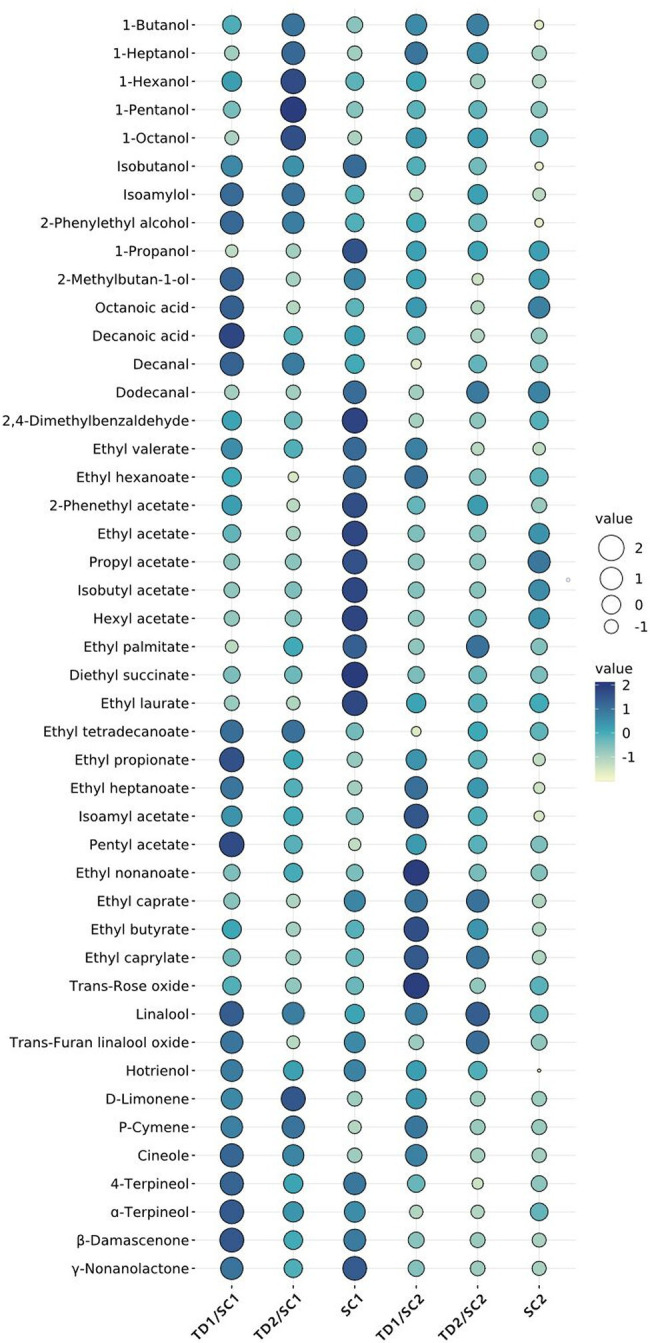
Bubble chart of major aroma compounds in pure and mixed fermented icewines. Size and color of bubble represent the relative abundance of major aroma compounds; the larger the size, the darker the color, the greater the relative abundance of aroma compound. TD1/SC1, TD2/SC1, TD1/SC2, TD2/SC2: sequential inoculation with *T. delbrueckii* (TD1, TD2) followed by *S. cerevisiae* (SC1, SC2) after 48 h; SC1 and SC2: single inoculation of *S. cerevisiae* SC1 and SC2, respectively.

Identification of volatile aroma compounds in pure and mixed culture fermented icewines was carried out by HS-SPME-GC-MS. A total of 45 major volatile aroma compounds are identified and listed in Table 2, which are related to the aroma of icewine. These volatile aroma compounds were divided into 6 classes, including 10 alcohols, 19 esters (7 acetate esters and 12 ethyl esters), 2 fatty acids, 3 aldehydes, 7 terpenes, 4 others. Meanwhile, Figure 2 demonstrates the relative abundance of major aroma compounds in the icewines fermented with different fermentation strategies, which is used to characterize the volatile compound distribution among each icewines.

**Table 2:** Major aroma compounds (mean GC-FID peak area × 10^6^) in mixed and pure culture fermented icewines.

Identity	RI^a^	CAS#	Odor descriptor^b^	Peak area^c^						Odorant series^d^
				TD1/SC1	TD2/SC1	SC1	TD1/SC2	TD2/SC2	SC2
***Alcohols***										
1-Propanol	597	71-23-8	Ripe fruit ^I^	0.03 ± 0^d^	0.05 ± 0^c^	0.21 ± 0.01^a^	0.12 ± 0^b^	0.12 ± 0.01^b^	0.13 ± 0^b^	1
1-Butanol	697	71-36-3	Fuseloil, sweet, balsam, medicinal ^II^	0.04 ± 0^d^	0.07 ± 0^a^	0.03 ± 0^e^	0.06 ± 0^c^	0.06 ± 0^b^	—	2,5
1-Pentanol	761	71-41-0	Balsamic ^III^	0.01 ± 0^a^	0.25 ± 0.32^a^	—	0.04 ± 0^a^	0.03 ± 0.01^a^	—	1, 3
1-Hexanol	860	111-27-3	Green, etherial, herbaceous ^I^	0.24 ± 0.01^b^	0.52 ± 0.01^a^	0.16 ± 0.01^d^	0.22 ± 0^c^	0.04 ± 0^e^	—	4
1-Heptanol	960	111-70-6	Intense citrus, oily ^IV^	—	0.21 ± 0^a^	—	0.19 ± 0.01^b^	0.14 ± 0.01^c^	—	1, 5
1-Octanol	1,059	111-87-5	Intense citrus, rose, jasmine, lemon ^V^	—	0.15 ± 0^a^	—	0.08 ± 0^b^	0.07 ± 0^c^	0.04 ± 0^d^	1,6
Isobutanol	597	78-83-1	Solvent, green, bitter, ethereal ^VI^	1.72 ± 0.02^a^^,^^b^	1.67 ± 0.04^a^^,^^b^	1.92 ± 0.01^a^	1.46 ± 0.09^b^	1.35 ± 0.03^b^	0.85 ± 0.49^c^	2
Isoamylol	697	123-51-3	Nail polish, alcohol ^VII^	18.68 ± 0.12^a^	17.94 ± 0.07^b^	11.92 ± 0.37^d^	6.25 ± 0.04^e^	13.58 ± 0.09^c^	5.92 ± 0.06^f^	2
Phenylethyl alcohol	1,136	60-12-8	Rose, honey ^VI^	12.15 ± 0.45^a^	8.12 ± 0.24^b^	6.72 ± 0.07^c^	6.92 ± 0.09^c^	6.48 ± 0.19^c^	4.21 ± 0.17^d^	6
2-Methylbutan-1-ol	697	137-32-6	Cheese, sweet ^III^	7.11 ± 0.01^a^	4.73 ± 0.17^d^	6.42 ± 0.53^b^	5.87 ± 0.06^c^	4.28 ± 0.04^e^	6.06 ± 0.18^b^^,^^c^	5
***Esters***										
Propyl acetate	686	109-60-4	Raspberry, pear ^VI^	—	—	0.08 ± 0^a^	—	—	0.06 ± 0^b^	1
Isobutyl acetate	721	110-19-0	Sweet, fruity, ethereal, banana, tropical ^VI^	0.07 ± 0^e^	0.08 ± 0^c^	0.31 ± 0.01^a^	0.08 ± 0^d^	0.07 ± 0^d^	0.19 ± 0^b^	1
Isoamyl acetate	820	123-92-2	Banana, fruity, sweet ^V^	5.13 ± 0.16^b^	4.69 ± 0.12^c^	4.24 ± 0.17^d^	6.25 ± 0.04^a^	4.61 ± 0.13^c^	3.12 ± 0.04^e^	1
Pentyl acetate	884	628-63-7	Sweet, fruity, banana ^VI^	—	0.05 ± 0.01^c^	0.14 ± 0^a^	0.07 ± 0^b^	0.04 ± 0^d^	0.05 ± 0^c^	1, 5
Hexyl acetate	984	142-92-7	Pleasant fruity, pear ^VI^	0.28 ± 0.03^d^	0.29 ± 0.01^c^^,^^d^	0.54 ± 0^a^	0.28 ± 0.02^c^^,^^d^	0.32 ± 0.02^c^	0.4 ± 0.01^b^	1
2-Phenethyl acetate	1,259	103-45-7	Roses, honey ^VI^	1.73 ± 0.02^b^	1.22 ± 0.07^e^	2.26 ± 0.08^a^	1.55 ± 0.04^c^	1.75 ± 0.13^b^	1.36 ± 0.04^d^	6
Ethyl acetate	586	141-78-6	Solvent, pineapple, pungent ^I^	9.68 ± 0.09^c^	6.83 ± 0.04^e^	17.44 ± 0.2^a^	8.59 ± 0.06^d^	8.43 ± 0.14^d^	12.2 ± 0.25^b^	3, 6
Ethyl propionate	686	105-37-3	Fruity, rum, etherial, pineapple ^III^	0.3 ± 0.01^a^	0.18 ± 0^c^	0.12 ± 0.01^e^	0.21 ± 0.01^b^	0.17 ± 0.01^d^	0.07 ± 0^f^	1
Ethyl butyrate	785	105-54-4	Apple, strawberry, banana ^VI^	0.33 ± 0^c^	0.24 ± 0.01^e^	0.31 ± 0.01^d^	0.49 ± 0^a^	0.36 ± 0.01^b^	0.22 ± 0.01^f^	1
Ethyl valerate	884	539-82-2	Sweet, apple, pineapple ^VI^	0.1 ± 0^b^	0.08 ± 0^c^	0.12 ± 0.01^a^	0.11 ± 0^b^	0.06 ± 0^d^	0.05 ± 0^d^	1
Ethyl hexanoate	984	123-66-0	Fruity, green apple, banana, brandy ^I^	2.55 ± 0.22^b^	1.75 ± 0.02^c^	3.09 ± 0.08^a^	3.06 ± 0.02^a^	2.25 ± 0.16^d^	2.46 ± 0.11^b^^,^^c^	1, 3
Ethyl heptanoate	1,083	106-30-9	Pineapple, fruity ^VI^	0.12 ± 0.01^a^	0.08 ± 0^c^	0.06 ± 0^e^	0.12 ± 0^a^	0.1 ± 0^b^	0.04 ± 0^d^	1
Diethyl succinate	1,151	123-25-1	Fruity, melon ^I^	—	0.03 ± 0^c^	0.46 ± 0^a^	—	0.04 ± 0^b^	—	1
Ethyl caprylate	1,381	110-38-3	Sweet, fruity ^IV^	2.75 ± 0.06^c^	1.74 ± 0.22^d^	2.92 ± 0.42^c^	6.42 ± 0.11^a^	5.41 ± 0.26^b^	1.31 ± 0.07^e^	1, 6
Ethyl nonanoate	1,282	123-29-5	Fruity, rose ^VI^	0.02 ± 0^c^	0.15 ± 0.12^b^	0.02 ± 0^c^	0.71 ± 0^a^	0.03 ± 0^c^	—	1, 6
Ethyl caprate	1,381	110-38-3	Fruity ^IV^	2.53 ± 0.13^c^	1.46 ± 0.08^d^	5.62 ± 0.14^b^	6.42 ± 0.2^a^	6.51 ± 0.13^a^	1.45 ± 0.1^d^	1, 6
Ethyl laurate	1,580	106-33-2	Sweet, floral, fruity ^VI^	0.36 ± 0.02^b^^,^^c^	0.26 ± 0.01^c^	1.52 ± 0.06^a^	0.79 ± 0.15^b^	0.67 ± 0.01^b^^,^^c^	0.73 ± 0.54^b^	1
Ethyl tetradecanoate	1779	124-06-1	Sweet, orris ^VI^	0.06 ± 0^a^	0.06 ± 0^a^	0.04 ± 0^c^	0.03 ± 0^d^	0.05 ± 0^b^	0.04 ± 0^b^^,^^c^	5
Ethyl palmitate	1978	628-97-7	Fruity ^VI^	0.04 ± 0^d^	0.08 ± 0^b^	0.11 ± 0.01^a^	0.06 ± 0.02^c^^,^^d^	0.11 ± 0^a^	0.06 ± 0^c^	1
***Acids***										
Octanoic acid	1,173	124-07-2	Rancid, vegetable ^VI^	0.33 ± 0.01^a^	—	0.12 ± 0^d^	0.2 ± 0.01^c^	—	0.25 ± 0.01^b^	5
Decanoic acid	1,372	334-48-5	Citrus, rancid sour ^VI^	0.39 ± 0.5^a^	0.13 ± 0.01^a^	0.18 ± 0^a^	0.11 ± 0^a^	—	0.05 ± 0^a^	5
***Aldehydes***										
Decanal	1,204	112-31-2	Rancid, intense citrus ^III^	0.29 ± 0.01^a^	0.28 ± 0.02^a^	0.25 ± 0^b^	0.19 ± 0.01^c^	0.24 ± 0.01^b^	0.23 ± 0.01^b^	2, 4
Dodecanal	1,402	112-54-9	Vanilla ^III^	—	—	0.02 ± 0^a^	—	0.02 ± 0^a^^,^^b^	0.01 ± 0^b^	2, 4
2,4-Dimethylbenzaldehyde	1,208	15,764-16-6	Cherry, almond, vanilla ^VI^	0.61 ± 0^b^	0.48 ± 0.02^e^	1.17 ± 0.02^a^	0.27 ± 0.02^d^	0.35 ± 0.03^f^	0.53 ± 0.01^c^	4
***Terpenes***										
trans-Rose oxide	1,114	876-18-6	Green, lychee, rose ^VI^	0.23 ± 0.02^b^	0.04 ± 0.01^e^	0.17 ± 0^d^	0.88 ± 0.01^a^	0.03 ± 0^e^	0.21 ± 0.01^c^	4, 5, 6
4-Terpineol	1,137	562-74-3	Peppery, lighter earthy ^VI^	0.59 ± 0.01^a^	0.5 ± 0.01^b^	0.56 ± 0.01^a^	0.46 ± 0.04^c^	0.37 ± 0.02^d^	0.43 ± 0.01^c^	4
α-Terpineol	1,143	98-55-5	Oil, anise, mint, lilac, floral, sweet ^I^	1.14 ± 0.01^a^	0.67 ± 0.01^b^	0.73 ± 0.54^a^^,^^b^	—	—	0.38 ± 0.28^b^^,^^c^	4, 6
Linalool	1,082	78-70-6	Floral, sweet, grape-like^I^	0.29 ± 0.01^c^	0.37 ± 0.01^a^	0.23 ± 0.01^d^	0.33 ± 0.01^b^	0.27 ± 0.01^c^	0.21 ± 0.03^d^	1, 6
D-Limonene	1,018	5,989-27-5	Citrus, orange, fresh, sweet ^VI^	0.11 ± 0^b^	0.17 ± 0.01^a^	—	0.09 ± 0^c^	—	—	1, 6
(E)-Furan linalool oxide	1,164	34,995-77-2	Woody, floral ^VI^	0.34 ± 0.03^a^	0.08 ± 0^d^	0.29 ± 0.01^b^	0.08 ± 0.02^c^	0.36 ± 0.46^a^	0.14 ± 0^c^	4, 6
Hotrienol	1,072	20,053-88-7	Sweet, tropical, fennel, ginger ^VI^	0.85 ± 0.03^a^	0.66 ± 0.02^c^	0.81 ± 0.01^b^	0.67 ± 0.02^c^	0.57 ± 0.01^d^	—	6
***Others***										
β-Damascenone	1,440	23,726-93-4	Apple, rose, honey, grape fruity, blueberry ^VI^	0.2 ± 0.01^a^	0.11 ± 0^c^	0.16 ± 0.01^b^	0.07 ± 0^d^	0.06 ± 0^e^	0.04 ± 0^f^	1, 5
P-Cymene	1,042	99-87-6	Fresh citrus, woody spice ^VI^	0.13 ± 0^b^	0.14 ± 0.02^a^	—	0.14 ± 0^a^^,^^b^	0.02 ± 0^c^	0.02 ± 0^c^	6
Cineole	1,059	470-82-6	Pine, camphor pungent, lavender oil ^VI^	0.59 ± 0.01^a^	0.44 ± 0.02^c^	0.02 ± 0^d^	0.46 ± 0^b^	—	—	6
γ-Nonanolactone	1,284	104-61-0	Coconut, peach, vanilla ^III^	0.11 ± 0^b^	0.08 ± 0^c^	0.12 ± 0^a^	0.06 ± 0^d^	0.05 ± 0^e^	0.05 ± 0.01^e^	1, 4

a *RI = Experimental retention index, which was determined on a capillary columnRxi™-5 ms*.

b *Reported odor descriptor: I. Peinado et al. (2004); II. Li et al. (2008); III. Fracassetti et al. (2019); IV. Sánchez-Palomo et al. (2010); V. Peinado et al. (2006); VI. http://www.thegoodscentscompany.com; VII. Siebert et al. (2005)*.

c *Different online roman letters in the same line show significant difference according to the Duncan test (*p* < 0.05)*.

d *1 = fruity; 2 = chemical; 3 = spicy; 4 = herbaceous; 5 = sweet; and 6 = flora*.

*“—” Indicates not detected*.

*Different lower-case letters represent significant differences*.

#### Alcohols

The higher alcohols, also known as fusel alcohols, are generated by the deamination of amino acids which is caused by living yeast cells during fermentation to meet protein requirements, *via* the Ehrlich pathway (Hazelwood et al., 2008). A total of 10 major alcohols were identified in pure and mixed fermented icewines (Table 2). The inoculation of *T. delbrueckii* affected the alcohols in icewines. 1-butanol, 1-hexanol, isoamylol and 2-phenylethyl alcohol in pure fermented icewines were lower levels than those in mixed culture fermented icewines, while 1-propanol was opposite. Interestingly, 1-pentanol and 1-heptanol were not found in pure fermented icewines, but they were detected in mixed culture fermented icewines.

#### Esters

Esters can influence the aroma of icewine through direct and complex synergistic interactions. Table 2 shows that a total of 19 representative esters including 7 acetate esters and 12 ethyl esters were identified in the icewines. The inoculation of *T. delbrueckii* affected the acetate esters in icewines. Propyl acetate, isobutyl acetate, hexyl acetate and ethyl acetate in pure fermented icewines were higher levels than those in mixed culture fermented icewines, while isoamyl acetate was opposite.

The ethyl esters were also affected by the inoculation of *T. delbrueckii*. In SC1 icewines, most ethyl esters in mixed culture fermented icewines (TD1/SC1 and TD2/SC1) were lower levels than those in pure fermented icewine (SC1). The interaction of *T. delbrueckii* and *S. cerevisiae* during mixed culture fermentation could enhance the production of ethyl propionate and ethyl heptanoate; moreover, in the icewines of TD1 involved (TD1/SC1 and TD1/SC2), the productions of these two ethyl esters were more than those of the icewines with TD2 participation (TD2/SC1 and TD2/SC2). The levels of ethyl caprate and ethyl caprylate were decreased in mixed culture fermentations with *T. delbrueckii* and SC1, while the increases of these two ethyl esters were found in mixed culture fermentation with *T. delbrueckii* and SC2; and the production of these two ethyl esters are largely linked to the biosynthesis of their acid precursors (octanoic acid and decanoic acid).

#### Acids and Aldehydes

Volatile acids are an important class of aromatic compounds. Octanoic acid and decanoic acid, which are medium-chain fatty acids (C6–C12), were detected in this study (Table 2). The highest levels of both compounds were found in TD1/SC1icewine, while the lowest levels were detected in TD2/SC2 icewine. In terms of aldehydes, dodecanal were not detected in mixed fermented icewines (TD1/SC1, TD2/SC1 and TD1/SC2). 2,4-Dimethylbenzaldehyde, which could negatively contribute to wine aroma due to its bitter almond note, in mixed fermented icewines was lower than that in pure *S. cerevisiae* fermented icewines (Table 2).

#### Terpenes and Others

The terpenes are usually present as non-volatile and non-aromatic compounds during wine fermentation which are complexed to glycosides and can be released by hydrolases, and contribute to wine aroma with significant influences on fruity and floral notes (López et al., 2014; Hong et al., 2021). The inoculation of *T. delbrueckii* affected the terpenes in icewines. In this study, linalool and D-limonene were lower levels in pure fermented icewines than those in mixed culture fermented icewines (Table 2). Trans-rose oxide, 4-terpineol, hotrienol and β-damascenone in icewines fermented with TD1 were higher levels than those in icewines fermented with TD2. Moreover, p-cymene and cineole were obviously increased in icewines of the sequential inoculation *T. delbrueckii/S. cerevisiae*.

#### Principal Component Analysis

**Figure 3:**
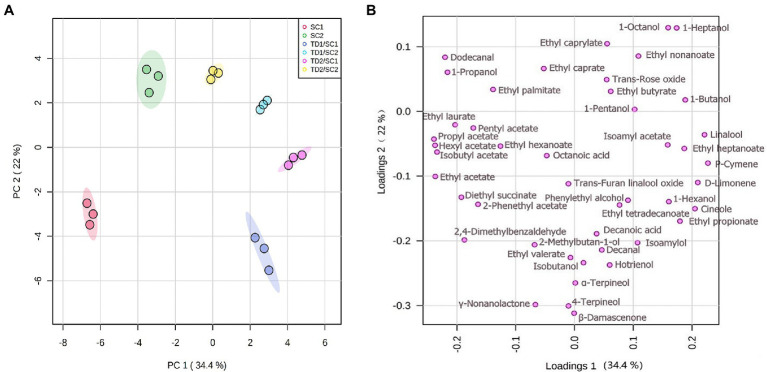
Principal component analysis (PCA) bi-plots of major aroma compounds in pure and mixed fermented icewines. There are score plot **(A)** and loading plot **(B)**. TD1/SC1, TD2/SC1, TD1/SC2, TD2/SC2: sequential inoculation with *T. delbrueckii* (TD1, TD2) followed by *S. cerevisiae* (SC1, SC2) after 48 h; SC1 and SC2: single inoculation of *S. cerevisiae* SC1 and SC2, respectively.

To highlight the major aromatic profiles of fermentations by different strategies and strains in icewines, the data of 45 major aromatic compounds were processed by PCA (Figure 3). The first two components (PCs) explained 56.6% of the variability, with PC1 and PC2 (34.4% and 22.2%, respectively). The icewines detected were clustered quite well, showing high experimental reproducibility. Based on the data obtained with PCA (Figure 3A), it should be remarked that the inoculation of *T. delbrueckii* indeed brought about significant differences on aromatic profile of icewines across PC1, especially in the case of mixed fermented icewines involving SC1; and the icewines fermented by the same *S. cerevisiae* strain could also be further separated from each other across PC2. According to Figure 3B, mixed fermented icewines of TD1/SC1 and TD2/SC1 were positioned in the lower right quadrant with higher level of positive higher alcohols, such as 2-phenylethyl alcohol and 1-propanol, and terpenes, such as β-damascenone, D-limonene, and linalool. Mixed fermented icewines of TD1/SC2 and TD2/SC2 were placed on the higher right quadrant mainly due to their higher levels of ethyl esters, such as, ethyl heptanoate, ethyl butyrate, and ethyl caprylate.

## Discussion

In this study, we demonstrated the growth dynamic changes of *T. delbrueckii* and the effect of mixed culture of *T. delbrueckii* and *S. cerevisiae* during fermentation on aroma compounds of Vidal blanc icewine, pure *S. cerevisiae* fermentation was used as the control. During mixed culture fermentations, *T. delbrueckii* gradually decreased and disappeared at different times, which might be ascribed to the competition for nutrients or cell–cell contact mechanisms during fermentations (Albergaria and Arneborg, 2016), or the ability of different *S. cerevisiae* strains to produce alcohol and the tolerance of specific *T. delbrueckii* strains toward ethanol (Hong et al., 2019).

Due to the inoculation of *T. delbrueckii* during fermentation, the basic chemical compositions of the icewines were affected, among which acetic acid was the most important to the quality of icewine. Acetic acid concentration was decreased, which is also consistent with the conclusion of Bely et al. (2008) and Comitini et al. (2011). Azzolini et al. (2015) pointed out that the reduction of acetic acid concentration can be extremely important to the results of sensory analysis of sweet wine. Some authors also reported *T. delbrueckii* as a low acetic acid producer compared with most non-*Saccharomyces* yeasts (Renault et al., 2009; Belda et al., 2015). Meanwhile, ethanol concentration was also decreased, which is consistent with the result of Belda et al. (2015). The reduction of acetic acid and ethanol concentration may be due to the consumption of sugar by *T. delbrueckii* for production of glycerol or pyruvic acid, or to an increase in yeast biomass, thus limiting the later *S. cerevisiae* fermentation activity (Kutyna et al., 2010; Belda et al., 2015; Lino et al., 2021). Nowadays, for consumer health and economic factors, as well as wine quality reasons, the wine sector is increasingly demanding technology to facilitate the production of wines with lower ethanol content (Kutyna et al., 2010; Contreras et al., 2014); the application of non-*Saccharomyces* yeasts during wine fermentation is one of the strategies. Several previous studies have shown that non-*Saccharomyces* yeasts usually have the effect of reducing ethanol content, reporting reductions higher than 1% in final ethanol concentration (Rantsiou et al., 2012; Contreras et al., 2014; Giaramida et al., 2016); in this study, it was reduced by about 2%. In order to combat the osmotic stress caused by the ice grape must, yeast cells may allocate carbon resources from sugar metabolism to produce metabolites necessary for adaptation and survival, of which glycerol is one (Heit et al., 2018; Lino et al., 2021). Glycerol is an important product of yeast fermentation, and its concentration in dry wine is usually 4–10 g/L, and higher glycerol levels are considered to improve wine quality (Heit et al., 2018). Regarding the increase in glycerol concentration, this is due to *T. delbrueckii* has a highly active glycerol-pyruvic pathway (Renault et al., 2009). In addition, differences in the fermentation kinetics among pure and mixed fermentations were evident (Supplementary Figure S1). Compared to the control fermentations, the lower fermentation kinetics of mixed culture fermentations were observed, which illustrated the fermentation kinetics appeared to be driven by the presence of *S. cerevisiae*.

In terms of higher alcohols, isoamylol and 2-phenylethyl alcohol are produced by yeasts during fermentation through conversions of leucine and phenylalanine *via* the Ehrlich pathway (Hazelwood et al., 2008). 2-Phenylethyl alcohol has pleasant notes with “honey,” “spice,” “rose,” and “lilac”; there was a relative increase of 2-phenylethyl alcohol in mixed fermented icewines, which is consistent with studies by Sun et al. (2014) in cherry wine, Chen and Liu (2016) in lychee wine and Zhang et al. (2018) in red wine. Higher isoamylol and 2-phenylethyl alcohol concentrations may be due to *T. delbrueckii* have preferences for leucine and phenylalanine consumption. 1-hexanol was associated with “vegetal” and “herbaceous” odor, which usually weaken the pleasant aroma quality of wines (Englezos et al., 2018); Sadoudi et al. (2012) and Zhang et al. (2018) demonstrated that 1-hexanol in wine fermented by *S. cerevisiae* was lower level than that in the wine of mixed culture fermented with *T. delbrueckii* and *S. cerevisiae*, which is consistent with the results of this study. However, 1-propanol, which usually has a positive influence on wine aroma with a ripe fruit note (Cai et al., 2014), was higher level in pure fermented icewines than in mixed culture fermented icewines. These results revealed that there may be some certain interaction between *S. cerevisiae* and *T. delbrueckii* for these alcohols, most probably a synergistic interaction (Sun et al., 2014). Moreover, 1-pentanol was associated with “balsamic” and “bitter almond” odor, and 1-heptanol has “oily” odor (Sánchez-Palomo et al., 2010); these two alcohols were apparently produced by *T. delbrueckii* participating during the icewine fermentation process. Besides, higher alcohols detected in mixed fermented icewines indicated that in addition to the diversity of *T. delbrueckii* strain, *S. cerevisiae* strain is an important factor in determining the formation of higher alcohols.

Esters are considered to provide positive contributions to wine’s fruity and flowery notes, which are produced by esterification of alcohols and acids at low pH (Saerens et al., 2010). Acetate esters are formed by condensation of higher alcohols with acetyl-coA, and catalyzed by ATF1 and ATF2 of alcohol acyl-transferases (AAT) genes in yeast cells (Peddie, 1990); they were thought to have a greater influence on wine aroma than ethyl esters (Lilly et al., 2000). In this study, we found that the inoculation of *T. delbrueckii* during mixed culture fermentation could promote the production of isoamyl acetate, which is consistent with studies by Renault et al. (2015). However, contradictory results were reported by Puertas et al. (2018). This may be due to the fact that *T. delbrueckii* was not totally displaced by *S. cerevisiae* in the study of Puertas et al. (2018), while *T. delbrueckii* was completely replaced by *S. cerevisiae* at the middle and later fermentative stages in the current study (Figure 1), and *T. delbrueckii* possessed a great hydrolytic activity of isoamyl acetate *via* esterase (Velázquez et al., 2015). Moreover, hexyl acetate has “apple,” “cherry,” and “pear” odor, propyl acetate has “celery” odor, and isobutyl acetate was associated with flowery fragrance (Hong et al., 2021). Ethyl acetate contributes to wine aroma with fruity note when the concentrations below 150 mg/L, otherwise it may negatively impart wine to nail polish remover odor (Swiegers et al., 2005). The effect of *T. delbrueckii* on these important volatile acetate esters seems to be pronounced, as demonstrated by the strong decline in ethyl acetate, isobutyl acetate and hexyl acetate in mixed fermented icewines. Similar results were previously reported (Sadoudi et al., 2012; Azzolini et al., 2015). This is mainly due to the reduction of acetic acid level by inoculation with *T. delbrueckii* (Lu et al., 2017). As for ethyl esters, they are formed by the reaction of ethanol with volatile fatty acid during the process of lipid biosynthesis (Saerens et al., 2010). Most ethyl esters in mixed culture fermented icewines were lower levels than those in pure fermented icewine; this is in agreement with the literature (Renault et al., 2015; Puertas et al., 2018).

In terms of fatty acids, the contribution of *T. delbrueckii* on wine aroma was clearly evident. The level of octanoic acid and decanoic acid in TD1 icewines were higher than those in icewines inoculated with pure *S. cerevisiae*, while the inoculation of TD2 could reduce the level of decanoic acid, and octanoic acid could not even be detected, which may be related to the production of their respective esters (Lu et al., 2017). Azzolini et al. (2015) pointed out that reductions in fatty acids can be considered positive as they are generally responsible for negatively affecting the overall wine aroma. Therefore, *T. delbrueckii* and *S. cerevisiae* mixed culture fermentation may be a strategy to modulate the production of ethyl esters and fatty acids in icewine.

As far as aldehydes are concerned, the sensory quality of decanal and dodecanal is generally considered to have no significant effect on wines (Bakker and Clarke, 2004). Dodecanal were not found in mixed fermented icewines, which might be due to their oxidation to corresponding acids or reduction to corresponding alcohols, and then conversion to related esters (Lu et al., 2017). Notably, 2, 4-dimethylbenzaldehyde was detected in icewine fermented by *T. delbrueckii* and *S. cerevisiae* for the first time.

Linalool is associated with flowery fragrance, and D-limonene has “citrus” and “fresh orange” odor. Linalool was increased in the icewines of sequential inoculation *T. delbrueckii*/*S. cerevisiae*, which is in agreement with recent studies (Azzolini et al., 2012; Zhang et al., 2018). The formation and levels of terpenes in mixed fermented icewines were evidently different, which may be related to the β-glucosidase activity of *T. delbrueckii* strains and terpene bioconversion rate (Sadoudi et al., 2012).

Notably, β-damascenone, which was described as providing sweet, exotic flower notes and honey flavor, has been reported as a key odorant in the aroma characteristics of Vidal icewine; small level changed of this metabolite can have a significant effect on sensory evaluation of icewine (Hong et al., 2021).

The results of pure fermentation showed that SC2 had different aromatic characteristic compared to SC1, for example, 1-hexanol, diethyl succinate and hotrienol were detected in SC1 pure fermented icewine, but not in SC2 pure fermented icewine; as expected, mixed culture of *T. delbrueckii* with SC1 or SC2 achieved distinct profiles of aromatic compounds. Meanwhile, there were significantly different levels in some aroma metabolites between the icewines fermented with TD1 and the icewines fermented with TD2, such as decanoic acid, D-limonene and cineole (Table 2; Figure 2); this result indicated that the strain-specificity of *T. delbrueckii* caused the different effects on icewine aroma metabolites.

In addition, the metabolic pathways of the main metabolites in icewine, such as ethanol, glycerol, organic acids, and terpenes, are more closely related to carbon sources. But, nitrogen is the main factor for yeast growth and fermentation activity, this parameter would be considered in our future research.

## Conclusion

To the best of our knowledge, this is the first study on the application of indigenous *T. delbrueckii* in Vidal blanc icewine fermentation, and volatile aroma compounds in the icewine fermented by *T. delbrueckii* and *S. cerevisiae*. The aromatic profiles of icewines under different fermentation strategies were distinct, which was significant for knowing more about the influences of *T. delbrueckii* on icewine quality. The inoculation of *T. delbrueckii* in icewine fermentation could decrease the concentration of acetic acid and ethanol, and increase the glycerol concentration; and produce more 2-phenylethyl alcohol, isoamyl acetate, linalool, D-limonene, p-cymene and cineole; and intensify the fruity, flowery, and sweet characteristic of icewine. Thus, the utilization of indigenous *T. delbrueckii* strains in mixed fermentation provided a potential way to improve aromatic complexity of icewines product and impart their unique regional flavor. Furthermore, the relevance of strain-specificity within *T. delbrueckii* to aroma compound differences was shown, which would provide insights for further investigations on the utilization of indigenous non-*Saccharomyces* yeasts strains in icewine making.

## Data Availability Statement

The original contributions presented in the study are included in the article/Supplementary Material; further inquiries can be directed to the corresponding author.

## Author Contributions

JL designed the experiments and wrote the manuscript. BQ analyzed the experimental data. MH conducted the experiments. All authors contributed to the article and approved the submitted version.

## Funding

The conducted research received funding from the Department of Education of Liaoning Province, under grant agreement no. JYTQN2020033.

## Conflict of Interest

The authors declare that the research was conducted in the absence of any commercial or financial relationships that could be construed as a potential conflict of interest.

## Publisher’s Note

All claims expressed in this article are solely those of the authors and do not necessarily represent those of their affiliated organizations, or those of the publisher, the editors and the reviewers. Any product that may be evaluated in this article, or claim that may be made by its manufacturer, is not guaranteed or endorsed by the publisher.

## Footnotes

^1^ http://www.bioinformatics.com.cn/

^2^ http://www.metaboanalyst.ca/

## Supplementary Material

Supplementary Figure S1 Accumulated production of CO_2_ during pure and mixed culture fermentations. TD1/SC1, TD2/SC1, TD1/SC2, TD2/SC2: sequential inoculation with *Torulaspora delbrueckii* (TD1, TD2) followed by *Saccharomyces cerevisiae* (SC1, SC2) after 48 h; SC1 and SC2: single inoculation of *S. cerevisiae* SC1 and SC2, respectively.

## Abbreviations

WL nutrient agar: Wallerstein laboratory nutrient agar
ITS region: Internal transcribed spacer region
HPLC: High-performance liquid chromatography
GC: Gas-chromatography
HS-SPME-GC-MS: Headspacesolidphase-microextraction-gas-chromatography-mass-spectrometry

## References

[ref1] Albergaria H. Arneborg N. (2016). Dominance of *Saccharomyces cerevisiae* in alcoholic fermentation processes: role of physiological fitness and microbial interactions. Appl. Microbiol. Biotechnol. 100, 2035–2046. doi: 10.1007/s00253-015-7255-0, PMID: 26728020

[ref2] Alessandria V. Giacosa S. Campolongo S. Rolle L. Rantsiou K. Cocolin L. (2013). Yeast population diversity on grapes during on-vine withering and their dynamics in natural and inoculated fermentations in the production of icewines. Food Res. Int. 54, 139–147. doi: 10.1016/j.foodres.2013.06.018

[ref3] Azzolini M. Fedrizzi B. Tosi E. (2012). Effects of *Torulaspora delbrueckii* and *Saccharomyces cerevisiae* mixed cultures on fermentation and aroma of Amarone wine. Eur. Food Res. Technol. 235, 303–313. doi: 10.1007/s00217-012-1762-3

[ref4] Azzolini M. Tosi E. Lorenzini M. (2015). Contribution to the aroma of white wines by controlled *Torulaspora delbrueckii* cultures in association with *Saccharomyces cerevisiae*. World J. Microbiol. Biotechnol. 31, 1–17. doi: 10.1007/s11274-014-1774-1 25388474

[ref5] Bakker J. Clarke R. J. (2004). Wine Flavour Chemistry. (1st edition). Oxford, UK: Blackwell Publishing Ltd.

[ref6] Belda I. Navascués E. Marquina D. Santos A. Calderon F. Benito S. (2015). Dynamic analysis of physiological properties of *Torulaspora delbrueckii*in wine fermentations and its incidence on wine quality. Appl. Microbiol. Biotechnol. 99, 1911–1922. doi: 10.1007/s00253-014-6197-2, PMID: 25408314

[ref7] Belda I. Ruiz J. Beisert B. Navascués E. Marquina D. Calderón F. (2017). Influence of *Torulaspora delbrueckii* in varietal thiol (3-sh and 4-msp) release in wine sequential fermentations. Int. J. Food Microbiol. 257, 183–191. doi: 10.1016/j.ijfoodmicro.2017.06.028, PMID: 28668728

[ref8] Bely M. Stoeckle P. Masnuef-Pomarède I. Dubourdieu D. (2008). Impact of mixed *Torulaspora delbrueckii*–*Saccharomyces cerevisiae* culture on high-sugar fermentation. Int. J. Food Microbiol. 122, 312–320. doi: 10.1016/j.ijfoodmicro.2007.12.023, PMID: 18262301

[ref9] Bowen A. J. Reynolds A. G. (2015). Aroma compounds in Ontario Vidal and Riesling icewines. I. Effects of harvest date. Food Res. Int. 76, 540–549. doi: 10.1016/j.foodres.2015.06.046, PMID: 28455036

[ref10] Cai J. Zhu B. Q. Wang Y. H. Lu L. Lan Y. B. Reeves M. J.. (2014). Influence of pre-fermentation cold maceration treatment on aroma compounds of cabernet sauvignon wines fermented in different industrial scale fermenters. Food Chem. 154, 217–229. doi: 10.1016/j.foodchem.2014.01.003, PMID: 24518336

[ref11] Cavazza A. Grando M. S. Zini C. (1992). Rilevazione della flora microbica di mosti e vini. Vignevini 9, 17–20.

[ref12] Chen D. Liu S. Q. (2016). Transformation of chemical constituents of lychee wine by simultaneous alcoholic and malolactic fermentations. Food Chem. 196, 988–995. doi: 10.1016/j.foodchem.2015.10.047, PMID: 26593581

[ref13] Comitini F. Gobbi M. Domizio P. Romani C. Ciani M. (2011). Selected non-*Saccharomyces* wine yeasts in controlled multistarter fermentations with *Saccharomyces cerevisiae*. Food Microbiol. 28, 873–882. doi: 10.1016/j.fm.2010.12.001, PMID: 21569929

[ref14] Contreras A. Hidalgo C. Henschke P. A. Chambers P. J. Curtin C. Varela C. (2014). Evaluation of non-*Saccharomyces* yeasts for the reduction of alcohol content in wine. Appl. Environ. Microbiol. 80, 1670–1678. doi: 10.1128/AEM.03780-13, PMID: 24375129 PMC3957604

[ref15] Crandles M. Reynolds A. G. Khairallah R. Bowen A. (2015). The effect of yeast strain on odor active compounds in riesling and Vidal blanc icewines. LWT-Food Sci. Technol. 64, 243–258. doi: 10.1016/j.lwt.2015.05.049

[ref16] Englezos V. Rantsiou K. Cravero F. Torchio F. Pollon M. Fracassetti D.. (2018). Volatile profile of white wines fermented with sequential inoculation of *Starmerella bacillaris* and *Saccharomyces cerevisiae*. Food Chem. 257, 350–360. doi: 10.1016/j.foodchem.2018.03.018, PMID: 29622221

[ref17] Englezos V. Torchio F. Cravero F. Marengo F. Giacosa S. Gerbi V.. (2016). Aroma profile and composition of Barbera wines obtained by mixed fermentations of *Starmerella bacillaris* (synonym *Candida zemplinina*) and *Saccharomyces cerevisiae*. LWT-Food Sci. Technol. 73, 567–575. doi: 10.1016/j.lwt.2016.06.063

[ref18] Fracassetti D. Bottelli P. Corona O. Foschino R. Vigentini I. (2019). Innovative alcoholic drinks obtained by co-fermenting grape must and fruit juice. Meta 9:86. doi: 10.3390/metabo9050086, PMID: 31052321 PMC6571751

[ref19] Giaramida P. Ponticello G. Maiol S. D. Squadrito M. Genna G. Barone E.. (2016). *Candida zemplinina* for production of wines with less alcohol and moreglycerol. South African J. Enol. Vitic. 34, 204–210. doi: 10.21548/34-2-1095

[ref20] Hazelwood L. A. Daran J. M. Maris A. J. Pronk J. T. Dickinson J. R. (2008). The Ehrlich pathway for fusel alcohol production: a century of research on *Saccharomyces cerevisiae* metabolism. Appl. Environ. Microbiol. 74, 2259–2266. doi: 10.1128/AEM.02625-07, PMID: 18281432 PMC2293160

[ref21] Heit C. Martin S. J. Yang F. Inglis D. L. (2018). Osmoadaptation of wine yeast (*Saccharomyces cerevisiae*) during icewine fermentation leads to high levels of acetic acid. J. Appl. Microbiol. 124, 1506–1520. doi: 10.1111/jam.13733, PMID: 29444384

[ref22] Hong M. Li J. Chen Y. (2019). Characterization of tolerance and multi-enzyme activities in non*-Saccharomyces* yeasts isolated from Vidal blanc icewine fermentation. J. Food Biochem. 43, 1–10. doi: 10.1111/jfbc.13027 31478209

[ref23] Hong M. Li J. Chen Y. Qi B. Y. Huang Y. P. Wu J.. (2021). Impact of mixed non-*Saccharomyces* yeast during fermentation on volatile aroma compounds of Vidal blanc icewine. LWT-Food Sci. Technol. 145:111342. doi: 10.1016/j.lwt.2021.111342

[ref24] Huang L. Ma Y. Tian X. Li J. M. Li L. X. Tang K.. (2018). Chemosensory characteristics of regional Vidal icewines from China and Canada. Food Chem. 261, 66–74. doi: 10.1016/j.foodchem.2018.04.021, PMID: 29739607

[ref25] Kutyna D. R. Varela C. Henschke P. A. Chambers P. J. Stanley G. A. (2010). Microbiological approaches to lowering ethanol concentration in wine. Trends Food Sci. Technol. 21, 293–302. doi: 10.1016/j.tifs.2010.03.004

[ref26] Lan Y. B. Qian X. Yang Z. J. Xiang X. F. Yang W. X. Liu T.. (2016). Striking changes in volatile profiles at sub-zero temperatures during over-ripening of ‘Beibinghong’ grapes in northeastern China. Food Chem. 212, 172–182. doi: 10.1016/j.foodchem.2016.05.143, PMID: 27374521

[ref27] Lan Y. B. Xiang X. F. Qian X. (2019). Characterization and differentiation of key odor-active compounds of ‘Beibinghong’ icewine and dry wine by gas chromatography-olfactometry and aroma reconstitution. Food Chem. 287, 186–196. doi: 10.1016/j.foodchem.2019.02.074, PMID: 30857688

[ref28] Lee S. B. Park H. D. (2020). Isolation and investigation of potential non-*Saccharomyces* yeasts to improve the volatile terpene compounds in Korean Muscat bailey a wine. Microorganisms 8:1552. doi: 10.3390/microorganisms8101552, PMID: 33050030 PMC7601120

[ref29] Lencioni L. Romani C. Gobbi M. Comitini F. Ciani M. Domizio P. (2016). Controlled mixed fermentation at winery scale using *Zygotorulaspora florentina* and *Saccharomyces cerevisiae*. Int. J. Food Microbiol. 234, 36–44. doi: 10.1016/j.ijfoodmicro.2016.06.004, PMID: 27367967

[ref30] Li J. Hu W. Z. Huang X. J. Xu Y. P. (2018). Investigation of yeast population diversity and dynamics in spontaneous fermentation of Vidal blanc icewine by traditional culture-dependent and high-throughput sequencing methods. Food Res. Int. 112, 66–77. doi: 10.1016/j.foodres.2018.06.011, PMID: 30131160

[ref31] Li J. Hu W. Z. Xu Y. P. (2019). Diversity and dynamics of yeasts during Vidal blanc icewine fermentation: a strategy of the combination of culture-dependent and highthroughput sequencing approaches. Front. Microbiol. 10:1588. doi: 10.3389/fmicb.2019.01588, PMID: 31354677 PMC6637317

[ref32] Li H. Tao Y. S. Wang H. Zhang L. (2008). Impact odorants of chardonnay dry white wine from Changli County (China). Eur. Food Res. Technol. 227(1), 287–292. doi: 10.1007/s00217-007-0722-9

[ref33] Lilly M. Lambrechts M. G. Pretorius I. S. (2000). Effect of increased yeast alcohol acetyltransferase activity on flavor profiles of wine and distillates. Appl. Environ. Microbiol. 66, 744–753. doi: 10.1128/AEM.66.2.744-753.2000, PMID: 10653746 PMC91891

[ref34] Lino F. Bajic D. Vila J. Sánchez A. Sommer M. (2021). Complex yeast–bacteria interactions affect the yield of industrial ethanol fermentation. Nat. Commun. 12:1498. doi: 10.1038/s41467-021-21844-7, PMID: 33686084 PMC7940389

[ref35] Liu P. T. Lu L. Duan C. Q. Yan G. L. (2016). The contribution of indigenous non-*Saccharomyces* wine yeast to improved aromatic quality of cabernet sauvignon wines by spontaneous fermentation. LWT-Food Sci. Technol. 71, 356–363. doi: 10.1016/j.lwt.2016.04.031

[ref36] Loira I. Vejarano R. Bañuelos M. A. Morata A. Tesfaye W. Tesfaye C.. (2014). Influence of sequential fermentation with *Torulaspora delbrueckii* and *Saccharomyces cerevisiae* on wine quality. LWT-Food Sci. Technol. 59, 915–922. doi: 10.1016/j.lwt.2014.06.019

[ref37] López S. Mateo J. J. Maicas S. (2014). Characterisation of *Hanseniaspora* isolates with potential aroma-enhancing properties in Muscat wines. South African J. Enol. Vitic. 35, 292–303. doi: 10.21548/35-2-1018

[ref38] Lu Y. Chua J. Y. Huang D. Lee P. R. Liu S. Q. (2017). Chemical consequences of three commercial strains of oenococcus oeni co-inoculated with *Torulaspora delbrueckii* in durian wine fermentation. Food Chem. 215, 209–218. doi: 10.1016/j.foodchem.2016.07.158, PMID: 27542469

[ref39] Maturano Y. P. Assaf L. A. R. Toro M. E. Nally C. Vallejo M. De Figueroa L. I. C.. (2012). Multi-enzyme production by pure and mixed cultures of *Saccharomyces* and non-*Saccharomyces* yeasts during wine fermentation. Int. J. Food Microbiol. 155, 43–50. doi: 10.1016/j.ijfoodmicro.2012.01.015, PMID: 22326141

[ref40] OIV (2010). Recueil international des méthodes d’analyse des vins et des moûts. Paris, France: Organisation Internationale de la Vigne et du Vin.

[ref41] OIV (2015). Icewine Oeno6/03. Available at: http://www.oiv.int/en/technical-standards-and-documents/products-definition-and-labelling/definition-of-the-vitivinicultural-products-by-code-sheet#i47

[ref42] Padilla B. Gil J. V. Manzanares P. (2016). Past and future of non-*Saccharomyces* yeasts: from spoilage microorganisms to biotechnologicaltools for improving wine aroma complexity. Front. Microbiol. 7:411. doi: 10.3389/fmicb.2016.00411 27065975 PMC4814449

[ref43] Peddie H. A. B. (1990). Ester formation in brewery fermentations. J. Inst. Brew. 96, 327–331. doi: 10.1002/j.2050-0416.1990.tb01039.x

[ref44] Peinado R. A. Mauricio J. C. Moreno J. (2006). Aromatic series in sherry wines with gluconic acid subjected to different biological aging conditions by *Saccharomyces cerevisiae* var. capensis. Food Chem. 94, 232–239. doi: 10.1016/j.foodchem.2004.11.010

[ref45] Peinado R. A. Moreno J. Bueno J. E. Moreno J. A. Mauricio J. C. (2004). Comparative study of aromatic compounds in two young white wines subjected to pre-fermentative cryomaceration. Food Chem. 84, 585–590. doi: 10.1016/S0308-8146(03)00282-6

[ref46] Puertas B. Jimenez-Hierro M. J. Cantos-Villar E. (2018). The influence of yeast on chemical composition and sensory properties of dry white wines. Food Chem. 253, 227–235. doi: 10.1016/j.foodchem.2018.01.039, PMID: 29502826

[ref47] Rantsiou K. Dolci P. Giacosa S. Torchio F. Tofalo R. Torriani S.. (2012). *Candida zemplinina* can reduce acetic acid produced by *Saccharomyces cerevisiae* insweet wine fermentations. Appl. Environ. Microbiol. 78, 1987–1994. doi: 10.1128/AEM.06768-11, PMID: 22247148 PMC3298173

[ref48] Renault P. Coulon J. de Revel G. Barbe J. C. Bely M. (2015). Increase of fruity aroma during mixed, *T. delbrueckii/S. cerevisiae* wine fermentation is linked to specific esters enhancement. Int. J. Food Microbiol. 207, 40–48. doi: 10.1016/j.ijfoodmicro.2015.04.037, PMID: 26001522

[ref49] Renault P. Miot-Sertier C. Marullo P. Hernández-Orte P. Lagarrigue L. Lonvaud-Funel A.. (2009). Genetic characterization and phenotypic variability in *Torulaspora delbrueckii* species: potential applications in the wine industry. Int. J. Food Microbiol. 134, 201–210. doi: 10.1016/j.ijfoodmicro.2009.06.008, PMID: 19619911

[ref50] Sadoudi M. Tourdot-Maréchal R. Rousseaux S. Steyer D. Gallardo-Chacón J. J. Ballester J.. (2012). Yeast–yeast interactions revealed by aromatic profile analysis of sauvignon Blanc wine fermented by single or co-culture of non-*Saccharomyces* and *Saccharomyces* yeasts. Food Microbiol. 32, 243–253. doi: 10.1016/j.fm.2012.06.006 22986187

[ref51] Saerens S. M. Delvaux F. R. Verstrepen K. J. Thevelein J. M. (2010). Production and biological function of volatile esters in *Saccharomyces cerevisiae*. Microb. Biotechnol. 3, 165–177. doi: 10.1111/j.1751-7915.2009.00106.x, PMID: 21255318 PMC3836583

[ref52] Sánchez-Palomo E. Gómez García-Carpintero E. Alonso-Villegas R. González-Viñas M. A. (2010). Characterization of aroma compounds of Verdejo white wines from the La Mancha region by odour activity values. Flavour Fragr. J. 25, 456–462. doi: 10.1002/ffj.2005

[ref53] Siebert T. E. Smyth H. E. Capone D. L. Neuwöhner C. Pardon K. H. Skouroumounis G. K.. (2005). Stable isotope dilution analysis of wine fermentation products by HS-SPME-GC-MS. Anal. Bioanal. Chem. 381, 937–947. doi: 10.1007/s00216-004-2992-4, PMID: 15660221

[ref54] Sun S. Y. Gong H. S. Jiang X. M. (2014). Selected non*-Saccharomyces* wine yeasts in controlled multistarter fermentations with *Saccharomyces cerevisiae* on alcoholic fermentation behaviour and wine aroma of cherry wines. Food Microbiol. 44, 15–23. doi: 10.1016/j.fm.2014.05.007, PMID: 25084640

[ref55] Swiegers J. H. Bartowsky E. J. Henschke P. A. Pretorius I. S. (2005). Yeast and bacterial modulation of wine aroma and flavour. Aust. J. Grape Wine Res. 11, 139–173. doi: 10.1111/j.1755-0238.2005.tb00285.x

[ref56] Tofalo R. Patrignani F. Lanciotti R. Perpetuini G. Schirone M. Di Gianvito P.. (2016). Aroma profile of montepulciano d’abruzzo wine fermented by single and co-culture starters of autochthonous *Saccharomyces* and non-*Saccharomyces* yeasts. Front. Microbiol. 7:610. doi: 10.3389/fmicb.2016.00610 27199939 PMC4848713

[ref57] Tronchoni J. Curiel J. A. Morales P. Torres-Pérez R. Gonzalez R. (2017). Early transcriptional response to biotic stress in mixed starter fermentations involving *Saccharomyces cerevisiae* and *Torulaspora delbrueckii*. Int. J. Food Microbiol. 241, 60–68. doi: 10.1016/j.ijfoodmicro.2016.10.017, PMID: 27756034

[ref58] Velázquez R. Zamora E. Álvarez M. L. Hernández L. M. Ramírez M. (2015). Effects of new *Torulaspora delbrueckii* killer yeasts on the must fermentation kinetics and aroma compounds of white table wine. Front. Microbiol. 6:1222. doi: 10.3389/fmicb.2015.01222 26579114 PMC4630308

[ref59] Zhang B. Q. Luan Y. Duan C. Q. Yan G. L. (2018). *Torulaspora delbrueckii* use of co-fermentation with two strains with different aromatic characteristic to improve the diversity of red wine aroma profile. Front. Microbiol. 9:606. doi: 10.3389/fmicb.2018.00606, PMID: 29674999 PMC5895779

