# Supplementary figures and images for: Impact of Torulaspora delbrueckii During Fermentation on Aromatic Profile of Vidal Blanc Icewine
Source: Front Microbiol. 2022 Jun 7;13:860128. doi: 10.3389/fmicb.2022.860128 (PMC9209767; doi:10.3389/fmicb.2022.860128)

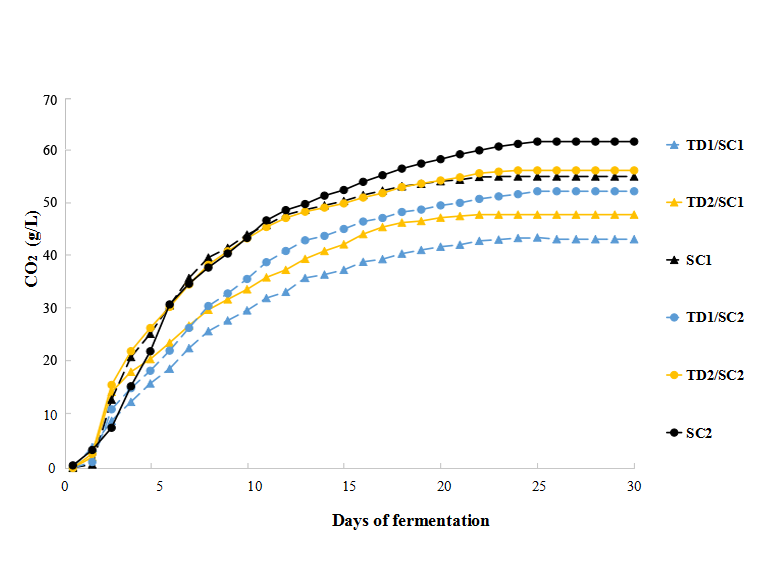

Supplement: Supplementary Figure S1 — Accumulated production of CO2 during pure and mixed culture fermentations. TD1/SC1, TD2/SC1, TD1/SC2, TD2/SC2: sequential inoculation with Torulaspora delbrueckii (TD1, TD2) followed by Saccharomyces cerevisiae (SC1, SC2) after 48 h; SC1 and SC2: single inoculation of S. cerevisiae SC1 and SC2, respectively. [file Image_1.TIF]
